# Cleavage Entropy as Quantitative Measure of Protease Specificity

**DOI:** 10.1371/journal.pcbi.1003007

**Published:** 2013-04-18

**Authors:** Julian E. Fuchs, Susanne von Grafenstein, Roland G. Huber, Michael A. Margreiter, Gudrun M. Spitzer, Hannes G. Wallnoefer, Klaus R. Liedl

**Affiliations:** Institute of General, Inorganic and Theoretical Chemistry, and Center for Molecular Biosciences Innsbruck (CMBI), University of Innsbruck, Innsbruck, Austria; University of Houston, United States of America

## Abstract

A purely information theory-guided approach to quantitatively characterize protease specificity is established. We calculate an entropy value for each protease subpocket based on sequences of cleaved substrates extracted from the MEROPS database. We compare our results with known subpocket specificity profiles for individual proteases and protease groups (e.g. serine proteases, metallo proteases) and reflect them quantitatively. Summation of subpocket-wise cleavage entropy contributions yields a measure for overall protease substrate specificity. This total cleavage entropy allows ranking of different proteases with respect to their specificity, separating unspecific digestive enzymes showing high total cleavage entropy from specific proteases involved in signaling cascades. The development of a quantitative cleavage entropy score allows an unbiased comparison of subpocket-wise and overall protease specificity. Thus, it enables assessment of relative importance of physicochemical and structural descriptors in protease recognition. We present an exemplary application of cleavage entropy in tracing substrate specificity in protease evolution. This highlights the wide range of substrate promiscuity within homologue proteases and hence the heavy impact of a limited number of mutations on individual substrate specificity.

## Author Summary

Proteases show a broad range of cleavage specificities. Promiscuous proteases as digestive enzymes unspecifically degrade peptides, whereas highly specific proteases are involved in signaling cascades. As a quantitative index of substrate specificity was lacking, we introduce cleavage entropy as a measure of substrate specificity of proteases. This quantitative score allows for straight-forward rationalization of substrate recognition by a subpocket-wise assessment of substrate readout leading to specificity profiles of individual proteases as well as an estimate of overall substrate promiscuity. We present an exemplary application of the descriptor ‘cleavage entropy’ to trace substrate specificity through the evolution of different protease folds. Our score highlights the diversity of substrate specificity within evolutionary related proteases and hence the complex relationship between sequence, structure and substrate recognition. By taking into account the whole distribution of known substrates rather than simple substrate counting, cleavage entropy provides the unique opportunity to dissect the molecular origins of protease substrate specificity.

## Introduction

Proteases catalyze cleavage of peptide bonds and are involved in virtually all fundamental cellular processes [1] turning proteases into central drug targets [2]. Far over 500 proteases with unique substrate cleavage patterns have been identified in the human genome [3]. These patterns reach from specificity for a single peptide to broad spectra of cleaved peptides. For instance, digestive enzymes are known to process a wide range of substrate sequences in contrast to proteases involved in signaling pathways cleaving only very distinct peptide bonds [1]. These signaling cascades include the blood-clotting cascade [4], apoptosis pathways [5] and regulatory activation steps of digestive proteases [6]. Specificity of a protease is determined by interactions in the protein-protein interface of protease and substrate. The spectrum of substrates to be cleaved is classified by subpocket-wise interactions following the convention of Schechter and Berger [7]: The peptide's scissile bond is designated between N-terminal P1 and C-terminal P1′. These subpocket indices are incremented over sequential amino acids. Protease interface residues are numbered accordingly over all subpockets Sn-Sn′, thus ensuring that interacting residues are indexed with the same number. Binding modes of processed polypeptides are highly similar due to the fact that the substrate is locked in an extended beta conformation within the protease binding site [8], [9]. This canonical conformation usually includes residues in the P3-P3′ substrate region, at most extended to P5, in serine protease elastase [10].

Cleavage specificity is generally originating from distinct molecular interactions between substrate and enzyme. Simple cleavage rules for serine proteases only rely on the prominent P1-S1 interactions. For instance, the hydrophobic S1 pocket of chymotrypsin causes specificity for substrates providing hydrophobic residues at their P1 position. In contrast, an Asp residue in the S1 site of the homologous trypsin determines specificity for Arg and Lys at P1 [11]. Limitations of such simple models are evident, as S1-directed mutation does not allow transposition of trypsin specificity to chymotrypsin [12]. Moreover, complex adjacent protein-loop interactions and dynamics were found to determine substrate specificity [13], [14].

Interactions between enzyme and substrate span several subpockets in the protease binding site. Experimental data shows that S2–S3 sites hardly affect substrate specificity in chymotrypsin [15], but account for specificity of the homologous elastase [16]. Especially chymotrypsin-like enteropeptidase shows exceptional specificity in the S5-S1-region cleaving only substrates containing the sequence Asp-Asp-Asp-Asp-Lys as trypsinogen [17]. P4-S4 interactions are found to be highly specific in case of the non-homologous subtilisin serine proteases [18]. Especially in the S1-S4-region, closely homologous serine proteases show significant differences in respective cleavage specificity reaching from limited proteolysis to almost unspecific substrate cleavage. Several cleavage site prediction tools are based on such simple and intuitive rules and are available online [19].

A plethora of experimental cleavage data for proteases is available in several databases. Cleavage information is generated experimentally by several methods reviewed by Diamond [20] and Poreba and Drag [21] reaching from fluorescence-based assays [22], isotopic labeling techniques [23], biotinylation schemes [24] over phage display [25], library-based approaches [26], microarray-based methods [27], [28] and combinations thereof to modern high-throughput techniques as proteomic identification of cleavage sites (PICS) [29], [30]. Cleavage data is accessible in several public databases including the MEROPS database [31], [32] linking structural protease data to cleavage activity.

Although cleavage information for known proteases is easily accessible, by now no attempt has been made to develop a quantitative measure for subpocket-wise and total protease specificity in contrast to pure feature extraction techniques as for example cascade detection [33]. Analysis of protease cleavage data was mostly limited to qualitative interpretation by conversion into consensus recognition motives and visualization by sequence logos [34], iceLogo [35] or heat maps [29]. We propose the usage of information entropy to merge experimental cleavage data into an easily interpretable score for subpocket specificity as well as overall protease specificity. Following the idea of information entropy [36], which is consistent with entropy in statistical mechanics [37], we developed an information theory-based specificity score named “cleavage entropy”. These cleavage entropy values depict a measure for uncertainty, and hence strictness of substrate readout, directly related to the information content of each amino acid position in a cleavage motif. A similar approach was successfully applied for description of sequence specificity of DNA binding proteins [38] and substrate promiscuity of whole enzyme families [39], including the P-region of proteases as an example [40]. DuVerle and Mamitsuka used information entropy for selection of a set of proteases showing diverse cleavage patterns and hence substrate promiscuity [41].

## Methods

### Extraction and Selection of Cleavage Data

To generate subpocket-wise specificity entropies, cleavage data were extracted from the MEROPS database [31]. Comparable cleavage databases as the CutDB [42] or Proteolysis MAP [43] were found to provide less cleavage information. Proteases of diverse families containing at least 100 substrate entries form a data set of 47 proteases. Methionyl aminopeptidases were excluded from the analysis, as positions P4-P2 remain unoccupied by the substrate upon cotranslational removal of N-terminal methionine residues. A complete sequence matrix containing the absolute occurrence of 20 amino acids at eight subpockets P4′ to P4 was compiled for each protease.

### Calculation of Subpocket-wise Cleavage Entropy

Protease-wise cleavage sequence matrices were normalized according to the natural abundance of individual amino acids [44]. Subsequently, a second normalization to 1 at each subpocket yielded a data matrix containing probabilities for each substrate amino acid at each protease subpocket. Information theory-based cleavage entropy is defined according to Formula 1 taking into account the whole distribution of amino acids at each position rather than a single peak of elevated amino acid abundance. Substrate information is purely incorporated as sequence, not covering any kind of secondary structure information. Derived dimensionless subpocket-wise entropy values, measure the broadness of distribution of cleaved substrates, range from 0 for a perfectly conserved single amino acid to 1 for an equal distribution of substrates, reflecting complete unspecific substrate binding.
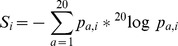
Formula 1: Calculation of subpocket-wise cleavage entropy S_i_ from subpocket-wise amino acid probabilities in known substrates p_a,i_.

### Calculation and Ranking According to Overall Cleavage Entropy

Subpocket-wise substrate specificity information is of high interest to compare individual subpockets of a single protease and individual specifity profiles between proteases. To facilitate analysis of different proteases as a whole, a summation of individual subpocket cleavage entropies yields quantitative overall cleavage entropy per protease (see Formula 2). This total cleavage entropy over eight substrate positions in the central binding site region (P4 to P4′) allows for ranking of proteases with respect to their whole substrate specificities. Entropy values range from 0 for a single conserved substrate to 8 for a random distribution of amino acids in cleaved substrates.
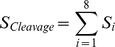
Formula 2: Calculation of overall protease cleavage S_Cleavage_ entropy by summation of 8 subpocket-wise cleavage entropies S_i_ from P4-P4′ subpockets.

### Cooperativity Effects in Substrate Readout: Pairwise Cleavage Entropy

Although cooperativity effects between subpockets were described for subtilisins [45] and reviewed by Ng et al. [46], available cleavage data only allows for a rough estimation of these correlation effects besides independent study of subpocket specificity. To cover inter-subpocket correlation effects in detail, data simply based on known substrates is too sparse. An extension from purely qualitative cleavage information to substrate-dependent quantitative binding affinity or kinetics measurements would be necessary. A suitable database containing diverse protease substrates is currently not known to the authors, but could also be of high interest to weight individual substrate contributions in order to refine the current implementation. A smaller set of fluorescence-based substrate turnover measurements for proteases was published by Harris et al. [22], but is restricted to variation of the P-region in substrates for eight proteases.

As only trypsin provides a sufficient data basis to study subpocket correlation effects with more than 14000 substrates listed in MEROPS, we performed an inter-subpocket correlation analysis only for this protease. The one-dimensional subpocket-wise cleavage entropy calculations presented above can directly be extended to a more-dimensional case yielding for two dimensions a pairwise cleavage entropy score depending on amino acids a and b at position i and j and their respective probabilities p_a,i_, p_b,j_.
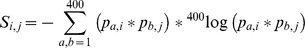
Formula 3: Calculation of pairwise cleavage entropy S_i,j_ from subpocket-wise amino acid pair probabilities in known substrates p_a,i_, p_b,j_.

This measure for inter-subpocket correlation effects yields as in the independent analysis (cleavage entropy) a score of 0 for a conserved single amino acid pair and a value of 1 for a distribution of amino acid pairs as expected by random chance from natural abundance [44]. To avoid artifacts from a lacking data basis we set a stringent cutoff of 10000 substrates in this two-dimensional analysis to allow for the same statistics as in the one-dimensional case (100 substrates).

### Phylogenetic Analysis of Protease Clans

As part of the discussion, protease specificity is compared to evolutionary distance. Sequences downloaded from Uniprot [47] as indexed in the MEROPS database [31] were grouped into respective protease clans. Sequences of each clan were sorted according to total cleavage entropy and aligned by ClustalW using default settings [48]. Tools from the EMBOSS server [49] were used for phylogenetic tree construction: fprotdist using default settings to calculate protein distance matrices, fkitsch using default settings to construct phylogenetic trees using the Fitch-Margoliash method [50]. Phylogenetic trees were visualized using Interactive Tree of Life (ITOL) [51].

### Visualization of Specificity Landscapes

Protein structure visualizations were created with PyMOL [52] based on the X-ray structures of trypsin and thrombin in complex with BIBR1109 (PDB: 1G32, 1G36) [53]. A subpocket definition derived from Bode et al. [54] was used for mapping of subpocket-wise cleavage entropies to the binding site region.

## Results

### Quantification of Subpocket-wise Cleavage Specificities

Entries with more than 100 annotated substrates in the MEROPS database represent 47 proteases comprise all major protease catalytic types. The three major protease catalytic types, serine, metallo and cysteine proteinases, covering more than 90% of known proteases [9], represent 40 entries or 85% of the test set. Calculated subpocket-wise cleavage entropies will be discussed by catalytic type to enable comparison of relative variation of binding specificity. Relative importance of subsites in determining cleavage specificity is highlighted by lowered entropy values providing specificity profiles for individual proteases.

**Figure 1:**
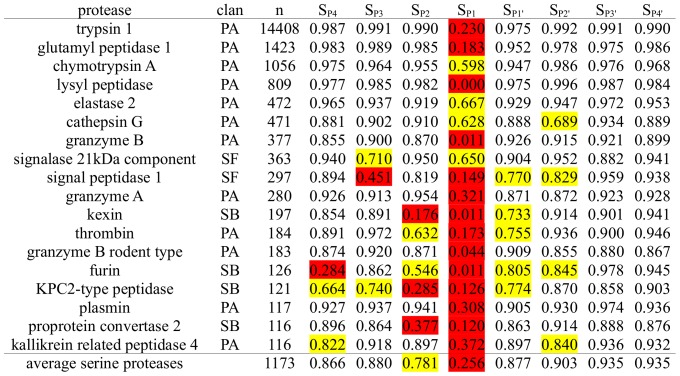
Subpocket-wise Cleavage Entropies of Serine Proteases. Serine proteases and associated MEROPS clans sorted according to the number of known substrates n with their respective subsite-wise cleavage entropies S_i_. Specific subpockets showing a cleavage entropy equal or less than an arbitrary cutoff of 0.85 are highlighted in yellow, values lower than 0.5 indicating stringent specificity in red.

**Figure 2:**
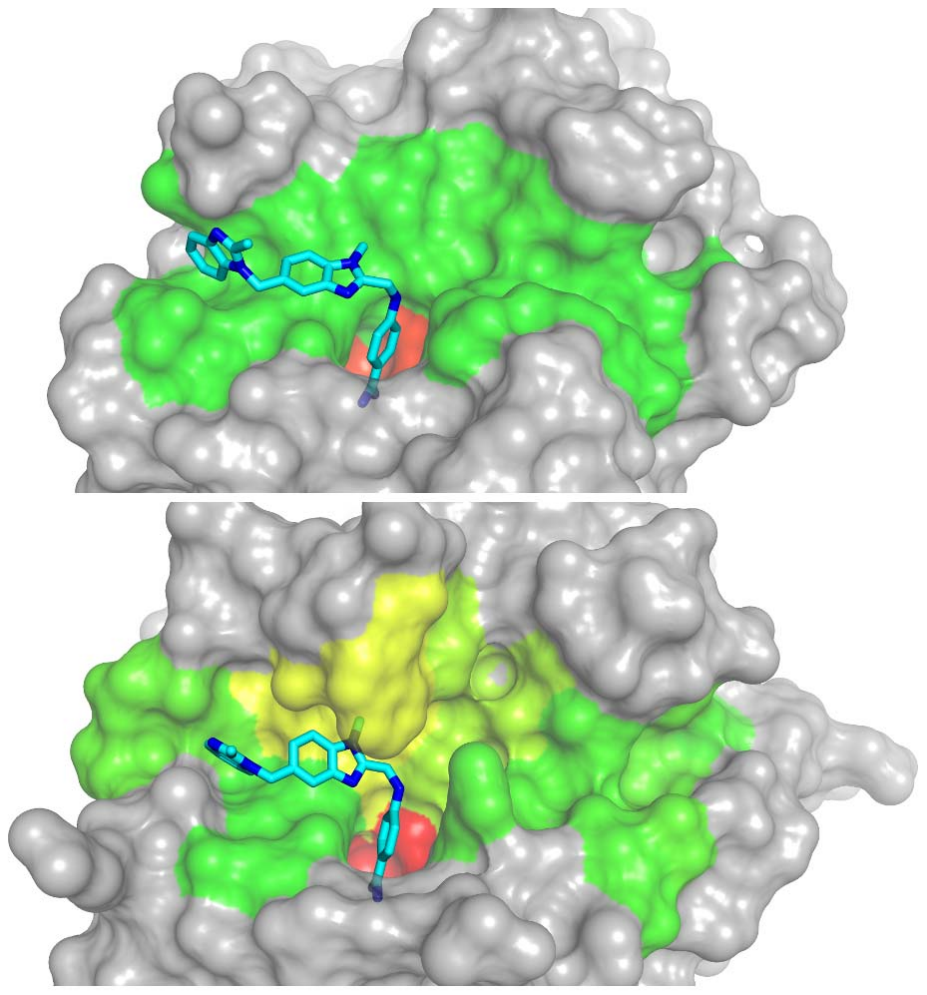
Specificity Landscapes of Trypsin and Thrombin. Subpocket-wise cleavage entropies mapped to the binding site region of trypsin (top) in a color spectrum of red (low, specific) over yellow to green (high, unspecific) highlight the central S1 pocket as only determinant of substrate specificity within the binding region S4-S4′ (left to right). By contrast, thrombin (bottom) binding the same small molecule inhibitor BIBR1109 [53] extends substrate recognition over further subpockets: the yellowish S1 and S1′ pockets in the specificity landscape of the binding site contribute to a more specific substrate readout.

Serine proteases show pronounced specificity at the P1 substrate site occupying the characteristic deep S1 pocket with an averaged cleavage entropy as low as S_P1_ = 0.256 (see Figure 1). The low P1 cleavage entropy value reflects widely accepted specificity rules for serine proteases solely based on P1-S1 interactions. A second hotspot for specific interactions of serine proteases is found in the P2-region with an average cleavage entropy of S_P2_ = 0.781, which is especially lowered for proprotein processing proteases kexin, furin and proprotein convertase 2 cleaving at paired basic residues [55]. Overall, serine proteases tend to bind conserved residues in P-region (average S_P4-P1_ = 0.696) rather than the P′-region (average S_P1′-P4′_ = 0.912) in accordance to findings of Page et al. for coagulation proteases as thrombin [56]. See Figure 2 for a detailed comparison of subpocket-wise cleavage entropies mapped to the three-dimensional structure of thrombin and trypsin.

All serine proteases in the test set show pronounced specificity in the P1-region, including even so-called unspecific proteases as trypsin binding to highly conserved arginine and lysine residues at the P1 site. An extension of this specific reading frame in both directions of the substrate is observed for example for thrombin and furin, where the latter protease shows extraordinary specificity at the P4 site independent of other specific residues. These lowered entropy values reflect the proposed Arg-Xaa-Lys/Arg-Arg consensus in the P4-P1-region for furin substrates [57] and confirm general specificity rules for P4 specificity of the subtilisin-like clan of serine proteases [18].

**Figure 3:**
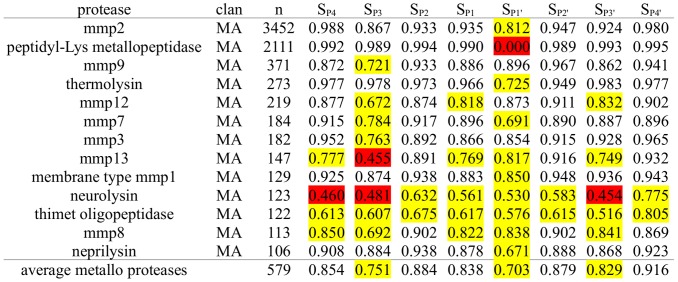
Subpocket-wise Cleavage Entropies of Metallo Proteases. Subpocket-wise cleavage entropies S_i_ of metallo proteases and associated MEROPS clans sorted by decreasing number of known substrates n. Specific pockets are highlighted in yellow and red according to their respective substrate promiscuity (yellow: 0.5<S_i_<0.85, red: S_i_<0.5).

Metallo proteases in general show less intense subpocket-wise specificity patterns than serine proteases. Their substrate readout is most pronounced in the P1′ position with an average cleavage entropy of 0.703 (see Figure 3) consistent with findings of Overall et al. for the substrate specificity of matrix metallo proteases [58]. Peptidyl-Lys metallo peptidase reads a perfectly conserved lysine residue at P1′ in all 2111 known substrates. However, P1′ is not the most specific subpocket in all metallo proteases. Further subpockets showing less pronounced substrate readout are located at P3 (S_P3_ = 0.751) and P3′ (S_P3′_ = 0.829) in analogy to computational predictions of Pirard [59]. Little substrate specificity is observed for other binding sites leading to an almost equivalent average substrate specificity over the whole P-and P′- region (S_P4-P1_ = 0.832, S_P1′-P4′_ = 0.831).

We find matrix metallo proteases (MMPs) to differ in their substrate specificity from other members of the metallo proteases. Cleavage entropy calculation highlights the P1′ position as major determinant of specificity in MMP-2, hence named “specificity pocket” [60], whereas other subsites show little substrate preferences. Additionally, a preference for proline at P3 has been observed [61], [62], which is consistent with lowered cleavage entropy values at P3 found throughout the MMP family. MMP-13 shows particular preference for proline residues at P3 reducing cleavage entropy to 0.455. Strikingly, particular metallo proteases span substrate specificity over all covered subsites: The highly specific members thimet oligopeptidase and neurolysin show cleavage entropy values lower than 0.850 throughout all subpockets.

**Figure 4:**
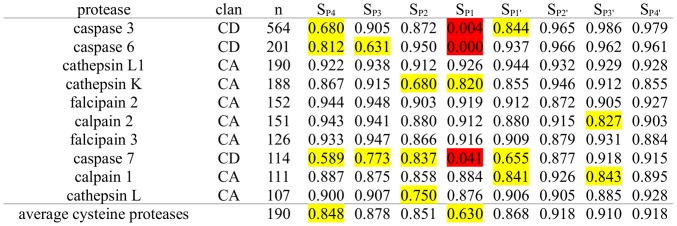
Subpocket-wise Cleavage Entropies of Cysteine Proteases. Cysteine proteases and associated MEROPS clans sorted according to the number of known substrates in MEROPS n. Subpocket-wise cleavage entropies S_i_ are color-coded to highlight specific pockets in yellow (0.5<S_i_<0.85). Highly specific subpockets are shown in red (S_i_<0.5).

Cysteine proteases are characterized by cleavage entropies comparable to serine proteasaes rather than metallo proteases. P1 interactions dominate substrate specificity with a cleavage entropy of S_P1_ = 0.630 similar to serine proteases (see Figure 4). Caspases account for the pronounced P1 interaction in this protease family as well as a smaller second specificity peak at P4 position (S_P4_ = 0.848). The P-region exhibits most of cysteine protease' substrate specificites with average cleavage entropy S_P4-P1_ = 0.802 compared to the P′-region S_P1′-P4′_ = 0.904.

Caspases are shown to read conserved aspartate residues in P1 position with an extraordinarily high specificity (P_1_<0.05), a characteristic not present in all other cysteine proteases. Subsite specificity of apoptosis signaling caspases [63] extends over larger areas of the P-region [64], especially pronounced in case of caspase 7 [29]. In contrast to caspases, calpains cleave broader substrate spectra whilst showing overlap with caspases in some regions of substrate space [65]. Traceable P3′ specificity is only observed for calpains amongst cysteine proteases. Broader distributions of substrates known for cathepsins [66] are quantitatively reflected by higher cleavage entropies. Cathepsin K's subtle substrate specificity at P1 and P1′ (S_P1_ = 0.680, S_P1′_ = 0.820) has been described by Schilling et al. [29]. Falcipains do not feature any particular subsite specificities, but tend to show complex and promiscuous specificity profiles. Simple counting of cleavage entries would have missed this unspecific behavior, as the number of available cleavage sites annotated in MEROPS is comparably low for falcipains.

**Figure 5:**
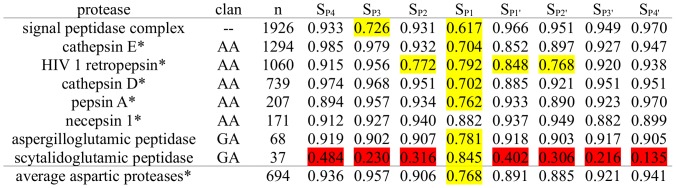
Subpocket-wise Cleavage Entropies of Further Proteases. Further proteases in the test set and associated MEROPS clans not belonging to the catalytic types cysteine, serine or metallo proteases sorted according to decreasing number of known substrates n. Specific subpockets (subpocket cleavage entropy 0.5<S_i_<0.85) are shown in yellow, highly specific pockets (S_i_<0.85) in red. Five aspartic proteases are marked with ‘*’.

Besides the three main classes of proteases, six further proteases with more than 100 cleavage patterns were found within MEROPS (see Figure 5): signal peptidase, containing a rare serine dyad at the active site [67], forming an active dimer complex in eukaryotes and hence indexed in MEROPS as complex peptidase, as well as five aspartic proteases. Two members of glutamic proteases showing distinct cleavage behavior were added to the sample to include this missing catalytic type, although less known cleaved peptides are indexed.

The signal peptidase complex is a membrane-bound protease involved in membrane translocation signaling [68]. Cleavage entropies S_P3_ = 0.726 and S_P1_ = 0.617 reflect the well-established specificity rules for signal peptidases focussing on positions P3 and P1 [69]. Distinct P1 specificity matches classical serine proteases involving a catalytic triad at the active site, whereas P3 readout is not a general characteristic of serine proteases.

All five aspartic proteases are found to depend mostly on P1 interactions with an average S_P1_ = 0.768. Other subpockets in P- and P′-region tend to exhibit likewise unspecific substrate binding (S_P4-P1_ = 0.892, S_P1′-P4′_ = 0.909). HIV retropepsin, a prominent target in drug design, shows distinct specificity at P2′ position with S_P2′_ = 0.768 supporting findings of Schilling et al. [29]. Furthermore, specific substrate readout of HIV retropepsin at positions P1 and P1′ was described in the literature [70] and is quantified with lowered cleavage entropies of S_P1_ = 0.792 and S_P1′_ = 0.848 respectively.

Aspergilloglutamic and scytalidoglutamic peptidase are added to the data set though sparse cleavage data to cover the group of glutamic peptidases represented by the members with highest number of annotated subtrates (68 and 37 respectively). Aspergilloglutamic and scytalidoglutamic peptidase provide two examples of variable cleavage profiles amongst the same protease class: Whereas the P1 position shows nearly identically lowered cleavage entropies, scytalidoglutamic peptidase reads substrate residues over the whole range of eight covered subpockets in contrast to aspergilloglutamic peptidase not showing pronounced substrate preferences at other subpockets than P1.

**Figure 6:**
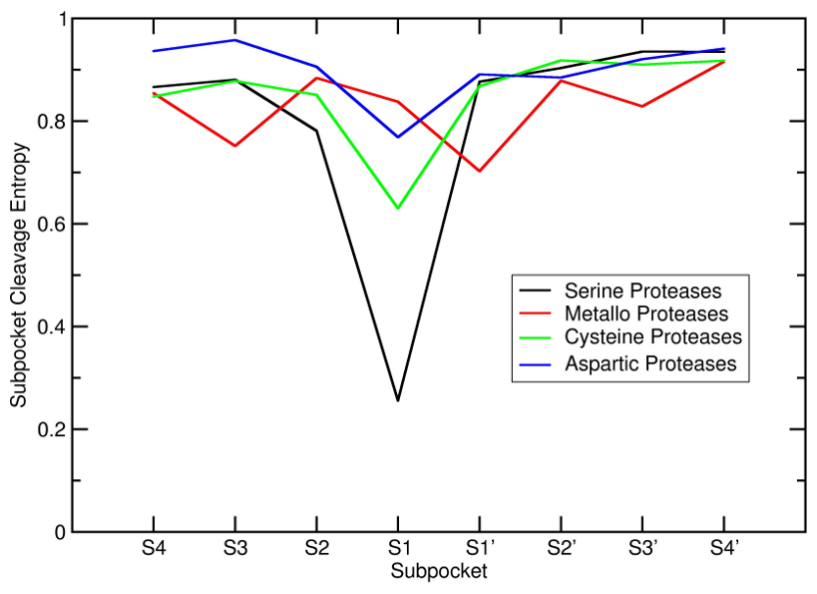
Subpocket-wise Cleavage Entropy Profiles for Protease Families. Subpocket-wise cleavage entropy profiles for protease catalytic classes reveal distinct substrate readout patterns for each of the protease groups. Serine proteases show most prominent subpocket specificity at the S1 site, whereas metallo proteases show specific binding behavior over a larger part of the binding pocket S4-S4′.

Summing up previous findings, average subpocket cleavage entropy profiles were calculated for protease catalytic types (see Figure 6). Serine proteases show distinct lowered cleavage entropy at their specific S1 site. Less pronounced S1 specificity is present for cysteine and aspartic proteases, whereas metallo proteases show subpocket cleavage entropy profiles including diverse cleavage entropy minima with the most specific substrate binding in the S1′ site.

### Ranking of Proteases According to Overall Cleavage Specificity

**Figure 7:**
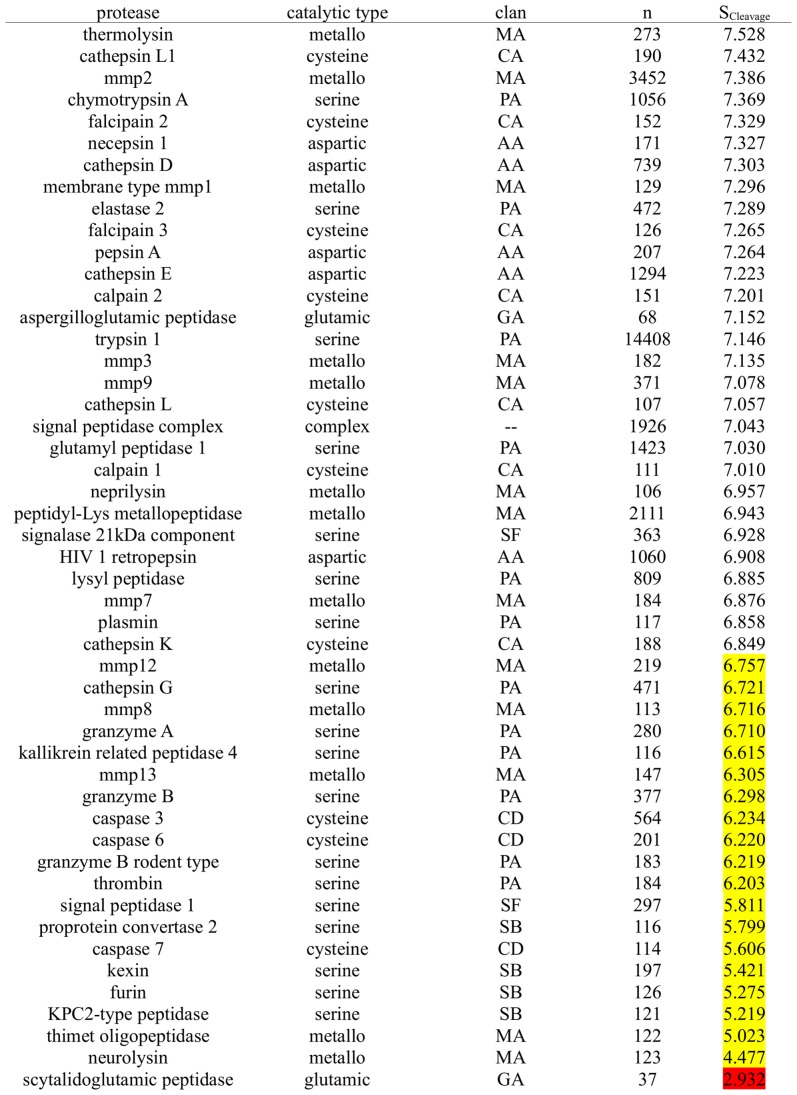
Total Cleavage Entropies of Investigated Proteases. Ranking of 49 proteases with respective MEROPS clan (including the added 2 glutamic proteases) in respect to their total cleavage entropy S_cleavage_. Specific proteases (S_Cleavage_<6.8, corresponding to an average subpocket cleavage entropy S_i_ of 0.85 over eight investigated subpockets)are highlighted in yellow. No protease in the core test set of 47 proteases is found to be highly specific (S_Cleavage_<4.0, reflecting an average S_i_ of lower than 0.5 over the whole binding site region of S_4_-S_4′_). Scytalidoglutamic peptidase present in the extended test set exhibits such strict substrate cleavage with a total cleavage entropy S_Cleavage_ of 2.932 owing to substrate recognition spreading over 7 highly specific subpockets (compare Figure 5).

Summation of subpocket-wise cleavage entropies yields a total estimate of protease specificity (see Figure 7). The additional information content of calculated total cleavage entropies compared to simple substrate counting is reflected by a squared linear correlation coefficient as low as r^2^ = 0.034 over the core test set of 47 proteases. Likewise, qualitative ranking correlation is comparably low with a Spearman ranking correlation of r = 0.334 over 47 proteases. Taking into account the whole distribution of amino acids in known substrates rather than the plain number of known substrates, has the advantage to minimize the impact of large scale profiling of closely related substrates biasing the underlying data set towards non-specificity. A second bias of the selected set of investigated proteases is thereby inevitable: the selection of peptidases with more than 100 annotated cleavage sites in MEROPS favors well-studied as well as unspecific proteases. Hence, a putative perfectly specific protease cleaving only a single substrate and hence, cleavage entropy of zero, would not be covered in the presented test set.

Proteases span a wide range of substrate specificites directly related to their biological roles. Ranking of the protease test set in respect to overall cleavage entropy S_Cleavage_ thus yields a clear separation between unspecific digestive proteases and specific proteases involved in signaling pathways. The protease with highest observed cleavage entropy S_Cleavage_ = 7.528, thermolysin, is involved in bacterial nutrition by unspecificly degrading exogenous peptides [71]. The technical usage in protein sequencing [72] and peptide synthesis [73] is facilitated by this unspecific substrate recognition of thermolysin. On the other end of the test set's specificity spectrum, neurolysin is a primary example for a specific signaling protease with S_Cleavage_ = 4.477. The limited proteolysis of intracellular oligopeptides by neurolysin [74] assures proper regulation of cell signaling [75].

### Cooperativity Effects Between Trypsin Subpockets

An exemplary analysis of inter-subpocket correlation was carried out based on over 14000 trypsin substrates listed in MEROPS (see Table S1). Only pairs including the specific P1 position show pronounced imbalances in two-dimensional distributions of substrate amino acid pairs reflected in lowered pairwise cleavage entropy scores. All other subpocket pairs show pairwise cleavage entropies in the range of 0.896 to 0.923 implying low correlation between subpocket readout. If at all a cooperative effect can be detected between P1′ and P2 in the underlying dataset for trypsin (S_P1′,P2_ = 0.896).

## Discussion

**Figure 8:**
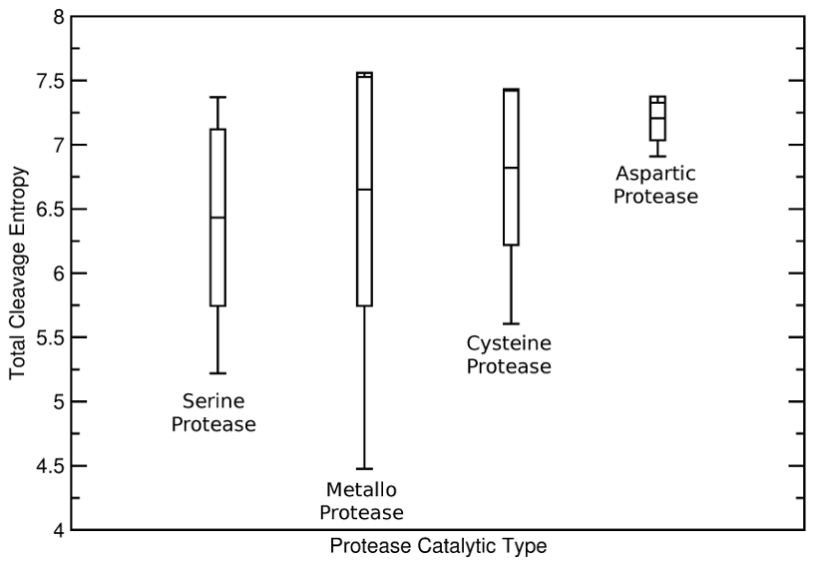
Total Cleavage Entropies of Protease Catalytic Types. Protease cleavage entropies indicate specific as well as unspecific members for each of the investigated protease catalytic machineries. As cleavage entropies (indicated by averages, maxima, minima and standard deviations) overlap between each of the types, the catalytic mechanism is found not to determine substrate specificity.

We proved cleavage entropy calculation as an intuitive approach to assess protease specificity quantitatively. In a first application of the presented score metric, we dissect the protease test set into groups of common cleavage machinery groups to elucidate potential descriptors of protease substrate specificity. This split yields four separate groups indicating distinct catalytic function: serine, metallo, cysteine and aspartic proteases (see Figure 8).

### Diversity of Substrate Readout Within Proteases Sharing a Catalytic Mechanism

Strikingly, both extrema on the presented quantitative protease specificity scale for the core set of 47 proteases represent members of the metallo proteases (thermolysin and neurolysin respectively). This indicates that the catalytic cleavage machinery cannot be the major determinant of substrate specificity. Similarly, serine proteases including the prominent digestive enzymes trypsin, chymotrypsin, elastase as well as signaling peptidases kexin and furin show diverse substrate specificity. Solely the smaller sample of five aspartic proteases shows predominantly unspecific cleavage behavior with an average total cleavage entropy of S_Cleavage_ = 7.205 compared to an average of S_Cleavage_ = 6.608 for the other catalytic types. Other protease classes do not show significant differences in their substrate specificity (serine proteases: average S_Cleavage_ = 6.433, metallo proteases: average S_Cleavage_ = 6.652, cysteine proteases: average S_Cleavage_ = 6.820). All protease types except for aspartic proteases therefore include specific as well as unspecific members. Thus, our study underlines the broadly accepted finding that protease substrate specificity is determined by subpocket interactions of the protease rather than directly at the catalytic site.

### Conserved Substrate Promiscuity of Proteases within Same Clan

**Figure 9:**
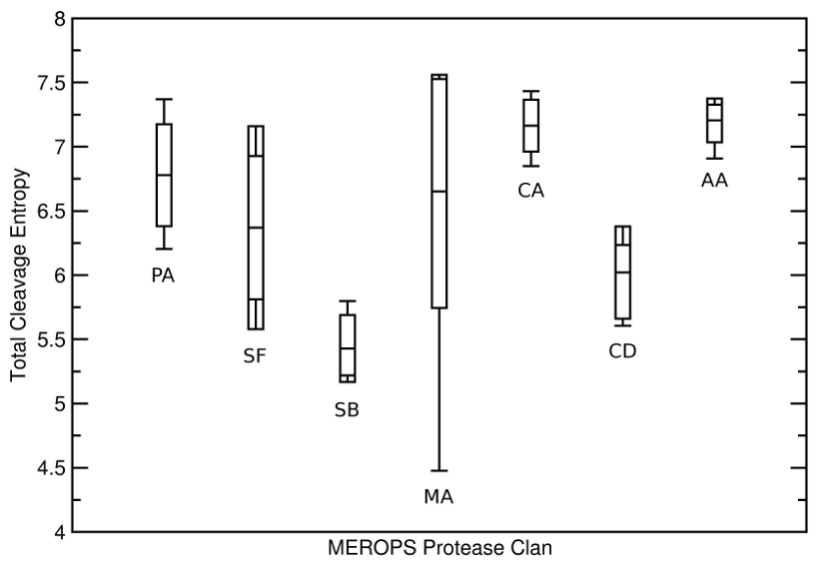
Total Cleavage Entropies of Protease Clans. Splitting of protease catalytic types into homologous protease clans allows to separate specific from unspecific members although they share a catalytic mechanism. Clan-wise total cleavage entropies are shown for MEROPS clans PA, SF, SB (all serine proteases), MA (metallo proteases), CA, CD (both cysteine proteases) and AA (aspartic proteases) with indicated averages, maxima, minima and standard deviations.

As apparent from Figure 8, the catalytic mechanism, does not discriminate specific from unspecific function. Rather, evolutionary related sub-groups sharing common catalytic mechanisms, but differing in three-dimensional fold are found to be similar in substrate promiscuity (see Figure 9). These clans within a catalytic class are not present in the test set for metallo proteases or aspartic proteases. All 13 metallo proteases in the test set belong to the MEROPS clan MA and all 5 aspartic proteases to the clan AA. Cysteine proteases spread over two distinct clans: 7 members (cathepsins, calpains and falcipains) belong to the CA papain clan, 3 others to clan CD, caspases. Serine proteases span three clusters of homologue proteases: 12 members are part of the PA clan (chymotrypsin-like proteases), containing besides serine proteases also cysteine proteases, that are not covered within the test set. Two members of the clan SF share the signalase fold, whilst four others share a subtilisin fold and thus belong to MEROPS clan SB. Signal peptidase complex is not assigned to a particular MEROPS protease clan.

Surprisingly, subdivision into homologue clans allows to subdivide proteases sharing the same catalytic mechanism into specific and unspecific subgroups. Cysteine proteases are divided into a more specific clan CD (average S_Cleavage_ = 6.020) and a relatively unspecific clan CA (average S_Cleavage_ = 7.163). Only caspases, known to be highly specific signaling proteases [76], represent clan CD in our test set, whereas calpains showing complex substrate specificities [41] with average S_Cleavage_ = 7.106, cathepsins with average S_Cleavage_ = 7.113 or falcipains with S_Cleavage_ = 7.297 are contained in clan CA. Falcipains of malaria-causing *Plasmodium falciparum* are involved in cytoskeleton and hemoglobin degradation [77] requiring unspecific substrate binding.

The same subdivision into specific and unspecific folds works for serine proteases that comprise clans of high specificity (clan SB: average S_Cleavage_ = 5.429), intermediate specificity (clan SF: average S_Cleavage_ = 6.370) as well as less specific proteases (clan PA: average S_Cleavage_ = 6.779). Standard deviations of cleavage entropies calculated within clan members are low (see Figure 9), suggesting intrinsically encoded limits for specific/non-specific behavior within the three-dimensional fold of the respective clans. This finding could be attributed to an intrinsic presence or absence of preorganized subpockets allowing for specific enzyme-substrate interactions.

Thus, the whole structure of protease clans has to be considered to shed light on the molecular origins of general protease cleavage spectra. Consistently, single mutations within specificity pockets of proteases are known to shift substrate spectra to other preferred substrates rather than to interchange specific and non-specific cleavage behavior. Nevertheless, a smooth interchange between specific and unspecific behavior including specialization and despecialization steps has been shown in case of granzymes [78], a class of serine proteases in clan PA.

### Rapid Evolutionary Interchange of Specificity within Protease Clans

**Figure 10:**
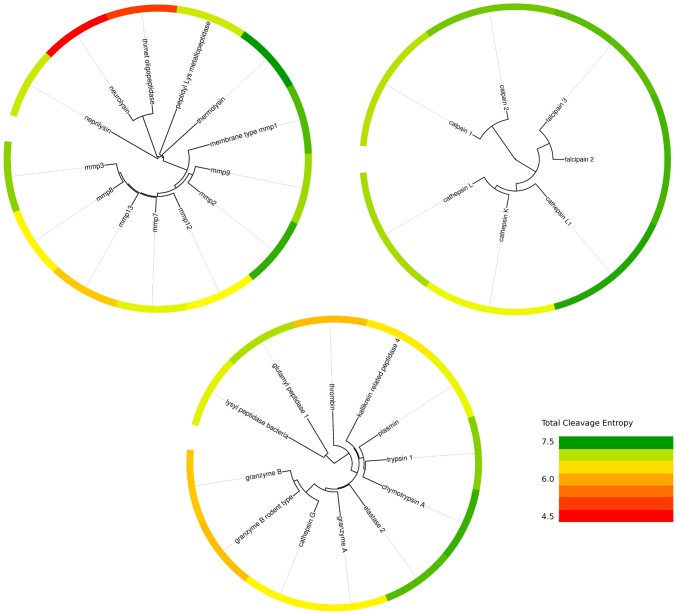
Phylogenetic trees of most prominent protease clans PA, CA and MA. Scattering of specific and unspecific behavior over respective evolutionary distances is apparent from the color-coded total cleavage entropy in all protease clans. Red fields indicate specific substrate recognition, whereas green fields mark unspecific proteases.

Further tracing the evolutionary development of protease specificity into particular protease clans arises the question, if evolutionary distance at sequence level is related to substrate specificity in these groups with conserved three-dimensional fold. Therefore, we performed a phylogenetic analysis for individual protease clans with more than five members contained in the test set (see Figure 10). MEROPS protease families are grouped in branches, confirming reasonability of presented phylogenetic trees. Whereas all members of clan PA belong to family S1, cysteine proteases spread over two distinct families: calpains are members of family C2 and are form a separate branch compared to all other proteases of the CA set that are part of the papain family C1. Metallo proteases belong to a wide-spread range of families: neprilysin is a singleton of family M13, neurolysin and thimet oligopeptidase of family M3 are nicely grouped in a separate branch. Two further singletons peptidyl-Lys metallo protease and thermolysin each form a separate tree branch for the families M35 and M4 respectively. All other members of clan MA are part of family M10, the matrix metallo peptidases, and are grouped into a broad branch separated from the other members.

Divergent evolution towards specific as well as unspecific members can be identified within all protease clans. Whereas a phylogenetic tree of metallo proteases of clan MA groups the highly specific members neurolysin and thimet oligopeptidase in a separate branch, indicating a close interplay between evolutionary distance and substrate specificity, this observation can not be extended to the whole set of proteases. The opposite holds even true in the MA clan for M10 family, where specific and unspecific members are grouped almost randomly compared to their evolutionary distance. The same complex behavior is found for cathepsins in clan CA: This branch includes the most specific member cathepsin L1 as well as the least specific member cathepsin K. Nevertheless, these members are grouped in closely related taxa indicating evolutionary proximity. Evolutionarily closely related proteases exhibit diverse substrate promiscuity in this protease group. Hence, protease evolution is capable of rapidly interchanging specific and non-specific substrate binding, implying a complicated relationship between protease sequence and substrate specificity.

The largest group of serine proteases of clan PA also groups specific and unspecific members in related taxa. E.g., cathepsin G and granzymes B of human and rodent origin exhibiting major different cleavage behavior are found as subbranch of closest evolutionary relation. Similarly, a branch including the rather specific signaling protease plasmin as well as the unspecific digestive enzymes trypsin 1 and chymotrypsin A, the most promiscuous members of this family, are grouped in close evolutionary proximity.

### Implications of the Evolution of Protease Specificity

We therefore surmise that a detailed understanding of protease specificity is only in reach within an even smaller subset of homologue proteases, where changes in substrate specificity can be attributed to a limited set of amino acid mutations, and hence atom exchanges, in the binding region. We propose to join forces between computational and experimental groups to elucidate structural hot-spots crucial for binding specificity in particular protease folds. According to the observed small fluctuations in specificity within respective clans, a smaller set of homologous proteases should be suitable to allow such in-depth investigations.

The presented specificity metric “cleavage entropy” for proteases can be applied to map subpocket-wise specificity contributions based on experimental data to individual subpockets of proteases as well as to calculate an estimate of overall substrate specificity. Furthermore, the extension of subpocket-wise cleavage entropies to pairwise cleavage entropies facilitates the detection of subpocket cooperativities in proteases provided that a sufficient number of substrates for this two-dimensional analysis is known. Thereby, drug design targeting proteases will profit from a thorough understanding of specific interactions to achieve desired protease selectivity [79] for example in targeting matrix metallo proteases [80]. As parameters at the level of sequence [81], structure [18] and conformational flexibility [82] are known to influence protease specificity, a direct quantification of substrate promiscuity of proteases will help to distinguish individual contributions to this phenomenon [83] and thereby support structural biology, the rational design of protease specificity [84] and the emerging field of degradomics [85]. An extension of the information-theory based specificity mapping towards general protein-protein interfaces to assess specificity and hence druggability of the respective interface regions is envisaged.

A straight-forward interpretable specificity score generally applicable to all families of proteases was presented that confirms widely accepted rules of thumb for protease cleavage in a quantitative way. Calculated cleavage entropies purely based on amino acid frequencies in known substrates allow a straight-forward assessment of subpocket-wise substrate specificities. According to our specificity metric, the catalytic cleavage machinery and thus, protease class, does not discriminate specific and unspecific proteases. In contrast, homologue protease clans share intrinsic specific and non-specific properties suggesting that protease specificity is encoded directly in the shared three-dimensional protein fold. Within particular protease clans and folds, a small number of mutations can cause drastic alterations of substrate specificity. These subtle changes at sequence, structure and flexibility level, but heavily impacting substrate promiscuity, are thus of high interest for structural biology but challenging to predict.

Unlike classical rules-of-thumb for protease specificity, the quantification of subpocket-wise and overall substrate specificity provides a continuous metric for specificity rather than a ‘yes’-or-‘no’ decision. The provided quantitative measure thus facilitates the comparison of the macromolecular descriptor “substrate specificity” with physicochemical, evolutionary and structural descriptors in protease recognition. Mapping of specificity to subpockets allows for intuitive visualization of structure-selectivity relationships in proteases and will thereby support the establishment of rules linking local protein structure and specificity.

## Supporting Information

Table S1 **Pairwise Cleavage Entropies of Trypsin.** Interdependence in substrate readout of trypsin subpockets P4-P4′ is reflected quantitatively as pairwise cleavage entropies S_i,j_. For comparison subpocket-wise cleavage entropies S_i_ are provided in the last row. Entropy values lower than 0.5 are highlighted in red, values between 0.5 and 0.85 in yellow. Besides readout of the P1 position, no pronounced cooperativity effect for trypsin can be observed.
(PDF)
