# Supplementary material for: Cleavage Entropy as Quantitative Measure of Protease Specificity
Source: PLoS Comput Biol. 2013 Apr 18;9(4):e1003007. doi: 10.1371/journal.pcbi.1003007 (PMC3630115; doi:10.1371/journal.pcbi.1003007)
Supplement: Table S1 — Pairwise Cleavage Entropies of Trypsin. Interdependence in substrate readout of trypsin subpockets P4-P4′ is reflected quantitatively as pairwise cleavage entropies Si,j. For comparison subpocket-wise cleavage entropies Si are provided in the last row. Entropy values lower than 0.5 are highlighted in red, values between 0.5 and 0.85 in yellow. Besides readout of the P1 position, no pronounced cooperativity effect for trypsin can be observed. (PDF) [file pcbi.1003007.s001.pdf]

Table S1:

Pairwise Cleavage Entropies of Trypsin: Interdependence in substrate readout of trypsin subpockets P4-P4' is reflected quantitatively as pairwise cleavage entropies $S_{i,j}$. For comparison subpocket-wise cleavage entropies $S_i$ are provided in the last row. Entropy values lower than 0.5 are highlighted in red, values between 0.5 and 0.85 in yellow. Besides readout of the P1 position, no pronounced cooperativity effect for trypsin can be observed.

| $S_{i,j}$ | P4 | P3 | P2 | P1 | P1' | P2' | P3' | P4' |
|---|---|---|---|---|---|---|---|---|
| P4 | | 0.909 | 0.911 | 0.537 | 0.904 | 0.911 | 0.920 | 0.923 |
| P3 | | | 0.907 | 0.537 | 0.902 | 0.923 | 0.913 | 0.920 |
| P2 | | | | 0.535 | 0.896 | 0.913 | 0.913 | 0.915 |
| P1 | | | | | 0.528 | 0.536 | 0.538 | 0.540 |
| P1' | | | | | | 0.900 | 0.903 | 0.914 |
| P2' | | | | | | | 0.918 | 0.922 |
| P3' | | | | | | | | 0.922 |
| P4' | | | | | | | | |
| $S_i$ | 0.987 | 0.991 | 0.990 | 0.230 | 0.975 | 0.992 | 0.990 | 0.990 |
